# Exploring biogeographic patterns of bacterioplankton communities across global estuaries

**DOI:** 10.1002/mbo3.741

**Published:** 2018-10-10

**Authors:** Anwesha Ghosh, Punyasloke Bhadury

**Affiliations:** ^1^ Integrative Taxonomy and Microbial Ecology Research Group, Department of Biological Sciences Indian Institute of Science Education and Research Kolkata Mohanpur‐741246 West Bengal India

**Keywords:** 16S rRNA, bacterioplankton, biogeography, estuaries, mangroves, next‐generation sequencing

## Abstract

Estuaries provide an ideal niche to study structure and function of bacterioplankton communities owing to the presence of a multitude of environmental stressors. Bacterioplankton community structures from nine global estuaries were compared to understand their broad‐scale biogeographic patterns. Bacterioplankton community structure from four estuaries of Sundarbans, namely Mooriganga, Thakuran, Matla, and Harinbhanga, was elucidated using Illumina sequencing. Bacterioplankton communities from these estuaries were compared against available bacterioplankton sequence data from Columbia, Delaware, Jiulong, Pearl, and Hangzhou estuaries. All nine estuaries were dominated by Proteobacteria. Other abundant phyla included Bacteroidetes, Firmicutes, Acidobacteria, Actinobacteria, Cyanobacteria, Planctomycetes, and Verrucomicrobia. The abundant bacterial phyla showed a ubiquitous presence across the estuaries. At class level, the overwhelming abundance of Gammaproteobacteria in the estuaries of Sundarbans and Columbia estuary clearly stood out amidst high abundance of Alphaproteobacteria observed in the other estuaries. Abundant bacterial families including Rhodobacteriaceae, Shingomonadaceae, Acidobacteriaceae, Vibrionaceae, and Xanthomondaceae also showed ubiquitous presence in the studied estuaries. However, rare taxa including Chloroflexi, Tenericutes, Nitrospirae, and Deinococcus‐Thermus showed clear site‐specific distribution patterns. Such distribution patterns were also reinstated by nMDS ordination plots. Such clustering patterns could hint toward the potential role of environmental parameters and substrate specificity which could result in distinct bacterioplankton communities at specific sites. The ubiquitous presence of abundant bacterioplankton groups along with their strong correlation with surface water temperature and dissolved nutrient concentrations indicates the role of such environmental parameters in shaping bacterioplankton community structure in estuaries. Overall, studies on biogeographic patters of bacterioplankton communities can provide interesting insights into ecosystem functioning and health of global estuaries.

## 1 INTRODUCTION

Bacterioplankton, a key component of the marine microbial loop, plays an important role in carbon and nitrogen cycles (Azam et al., 1983). Variations in hydrological parameters such as tidal inflow, salinity, and availability of organic matter may lead to strong environmental gradients for resident microbial communities (Herlemann et al., 2011; Hewson & Fuhrman, 2004; Kirchman, Dittel, Malmstrom, & Cottrell, 2005). Coastal ecosystems, such as estuaries, which are one of most productive ecosystems in the world (Kan, Suzuki, Wang, Evans, & Chen, 2007), represent an ideal niche to study bacterioplankton community structure and its associated functions. Continuous mixing of freshwater from the riverine and marine water makes estuaries highly dynamic ecosystems. Short residence time of water coupled with slow growth rates deter free‐living bacterial communities to efficiently develop into typical estuarine communities (Crump, Armbrust, & Baross, 1999). Previous studies have shown that estuarine bacterial communities harbor a mix of taxa that essentially belong to either freshwater or marine ecosystems (Crump et al., 1999; Herlemann et al., 2011). Functionally, bacteria in estuaries play an important role in the degradation of terrestrial and riverine organic carbon (Lee & Wakeham, 1988). In estuaries with low primary production, allochthonous carbon can be an important factor in supporting the food web by processes such as bacterial secondary production and through trophic transfers as part of the microbial loop (Murrell, Hollibaugh, Silver, & Wong, 1999). In coastal ecosystems such as estuarine mangroves, about 50% of the average litterfall is exported out of the ecosystem and eventually degraded by microbial communities including bacteria (Alongi, 2014). These organically rich mangroves form “specialized ecosystems” where resident bacterioplankton could be a key player in carbon cycling (Bano et al., 1997; Bianchi & Bauer, 2011).

Bacterioplankton community structure from various major estuaries such as the Chesapeake Bay (Bouvier & del Giorgio, 2002), Delaware estuary (Campbell & Kirchman, 2013; Kirchman et al., 2005), Columbia estuary (Fortunato & Crump, 2011), and Pearl estuary (Zhang, Jiao, Cottrell, & Kirchman, 2006) among others has been investigated using both Sanger and next‐generation sequencing (NGS) approaches. These studies have shown the presence of diverse bacterial phyla such as Proteobacteria, Actinobacteria, Bacteroidetes, Deferribacteres, and Verrucomicrobia across these sites. Furthermore, these studies have also shown spatial variation in bacterial classes such as Betaproteobacteria and Gammaproteobacteria and bacterial orders such as Burkholderiales, Rhodocyclales, Hydrogenophilales, and Methylophilales. This has broadened our understanding of bacterioplankton community structure and at the same time hinted toward difficulties in understanding habitat dynamics. Bacterioplankton communities from estuaries such as those located within mangrove ecosystems have rarely been looked into using uncultured approaches (Angsupanich, Miyoshi, & Hata, 1989; Ghaderpour et al., 2014; Gonsalves, Nair, LokaBharathi, & Chandramohan, 2009), and most of the studies have been restricted to soil and sedimentary habitats (Alfaro‐Espinoza & Ullrich, 2015; dos Santos et al., 2011; Ghosh et al., 2010; Gomes, Cleary, Calado, & Rodrigo, 2011) Due to the organic materials originating from litterfall, estuaries located within mangrove environments could potentially harbor novel bacterial groups. Therefore, undertaking survey of different estuaries to determine which microbial taxa are present where, and how and why they assemble into functional communities is integral to understanding the exact role of marine microbial communities in ecosystem processes.

The advent of NGS methods has allowed for increased sampling depth both in terms of number of sites covered and number of sequences generated per sample (Kircher & Kelso, 2010). Owing to restricted length of the sequenced amplicon, choice of efficient variable regions for taxonomic classification and phylogenetic analyses is still debated (Yang, Wang, & Qian, 2016). This could be circumvented by the introduction of Illumina sequencers such as MiSeq which can generate reads of up to 600 bp, thereby increasing the accuracy of downstream data processing (Degnan & Ochman, 2012). Work undertaken by Hao and Chen (2012) has shown that fragments of 16S ribosomal ribonucleic acid (16S rRNA) with length >150 bp from different portions of the marker gene could provide taxonomic assignment as accurately as the entire 16S rRNA sequence.

Such sequencing technologies have improved elucidation of bacterioplankton community structures from different ecosystems. Previous studies such as the ones highlighted below have shown spatial differences in many bacterial groups (Schauer, Massana, & Pedrόs‐Aliό, 2000; Ma et al., 2016; references herein). At lower taxonomic levels, bacteria representing the classes such as Alpha‐ and Betaproteobacteria have been found to be more abundant across marine and freshwater ecosystems, respectively (Cottrell & Kirchman, 2000; Glöckner, Fuchs, & Amann, 1999; Methe & Zehr, 1999; Tang et al., 2015). The abundance of members belonging to Alphaproteobacteria tends to be higher in environments with higher salinity (Cottrell & Kirchman, 2003). To date, reasons behind such observed biogeographic patterns of only certain groups of bacteria are not well understood. In light of such information, we hypothesize that bacterioplankton groups could exhibit distinct distribution patterns across estuaries under the influence of similar environmental parameters.

Coastal estuaries such as those located within mangroves ecosystems are particularly important from the viewpoint of organic carbon inputs (Dittmar, Hertkorn, Kattner, & Lara, 2006). Plant matter including litter fall contributes substantially to the pool of dissolved organic matter in such estuaries (Kristensen, Bouillon, Dittmar, & Marchand, 2008; Sardessai, 1993). Extensive studies have provided a comprehensive picture of the bacterioplankton communities from various global estuaries, while our knowledge of bacterioplankton communities of Sundarbans remains limited (Ghosh & Bhadury, 2017, 2018 ). Sundarbans is the largest contiguous stretch of mangrove forest globally that lies in the Ganga‐Brahmaputra‐Meghna (GBM) delta (Gopal & Chauhan, 2006). This ecosystem stretches over approximately 10,000 km^2^ spanning across India and Bangladesh and is characterized by the presence of perennial and rain‐fed rivers that open into the Bay of Bengal forming broad estuaries (WCMC, 2005). Nutrient‐rich coastal water flows in from the Bay of Bengal on a daily basis due to diurnal tidal action and mix with freshwater from these large rivers (Rahaman et al., 2013). High seasonal precipitation along with diurnal intrusion of tidal water rapidly changes the salinity profile of estuaries located in the Sundarbans (Choudhury & Bhadury, 2015; Rahaman et al., 2013).

The goal of this study was to examine biogeographic patterns of bacterioplankton communities from geographically distant estuaries. The objectives of this study were (a) to explore biogeographic patterns of bacterioplankton communities across major estuaries in comparison with the Sundarbans and (b) to understand the role of environmental parameters that lead to observed biogeographic patterns at broad phylogenetic levels.

## 2 MATERIALS AND METHODS

### 2.1 Sampling stations of estuaries in Sundarbans

**Figure 1:**
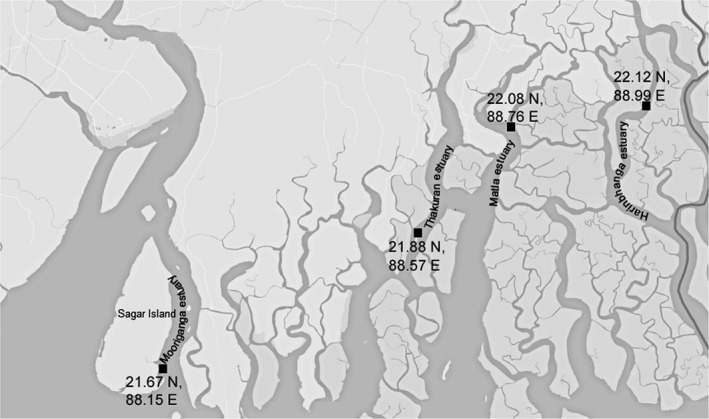
Map showing the location of four estuaries targeted from the Sundarbans mangrove ecoregion. The coordinates of the estuaries are Mooriganga estuary (21° 40ʹ N, 88° 09ʹ E), Thakuran estuary (21° 53ʹ N, 88° 34ʹ E), Matla estuary (22° 05ʹ N, 88° 45ʹ E), and Harinbhanga estuary (22° 07ʹN, 88° 59ʹ E)

Four stations located across four estuaries in Sundarbans selected for this study are shown in Figure 1. Sagar Island is the biggest island of Indian Sundarbans and marks the westernmost part of this ecoregion. A predesignated station named as Stn3 (Mooriganga) (21° 40ʹ 40.6ʺ N, 88° 09ʹ 19.2ʺ E) which is part of the Sundarbans Biological Observatory Time Series (SBOTS) monitored since 2010 has been considered in this study (Bhattacharjee, Samanta, Danda, & Bhadury, 2013; Choudhury, Das, Philip, & Bhadury, 2015; Samanta & Bhadury, 2014). Huge freshwater from the Mooriganga estuary and diurnal tidal influx of coastal water from the Bay of Bengal gives rise to typical estuarine conditions in this station. Moreover, this station is strongly influenced by local as well as regional precipitation caused by the southeastern monsoons (Gopal & Chauhan, 2006). Owing to its location near a densely human populated Sagar island, this station is subjected to intense anthropogenic activities including from agriculture fields and aquaculture farms. Additionally, one station each namely SBR_S1 (21° 53ʹ 21.99ʺ N, 88° 34ʹ 38.76ʺ E), SBR_S2 (22° 05ʹ 15.70ʺ N, 88° 45ʹ 55.60ʺ E), and SBR_S3 (22° 07ʹ 28.59ʺ N, 88° 59ʹ 48.39ʺ E) representing the other three estuaries, namely Thakuran estuary, Matla estuary, and Harinbhanga estuary, respectively, was considered in this study. These three estuaries are located in the central sector of the Indian Sundarbans. Due to heavy siltation, these three rivers have lost their upstream connection with the River Ganga and are only tidally fed on a diurnal basis (Banerjee, 2013; Manna, Chaudhuri, Bhattacharyya, & Bhattacharyya, 2010). Moreover, these three estuaries are located within the Sundarbans Biosphere Reserve (SBR) which is considered to be relatively pristine part of Sundarbans (Gopal & Chauhan, 2006). The SBR stations are located within the Sundarbans Biosphere Reserve, which is a heavily protected area and is a “high‐risk zone” for undertaking sampling work. Out of many stations that are usually sampled along these estuaries, one station (as mentioned above) each was selected for this study based on the comparable environmental parameters recorded during sampling. Stations along these estuaries vary largely from one another in salinity and water depth.

### 2.2 Sampling

One liter of surface water was collected from four estuaries (one station each) of Sundarbans following published protocol (Choudhury et al., 2015) and immediately fixed with molecular grade ethanol (Merck, Germany). Water was collected from Stn3 (Mooriganga) of SBOTS in July 2014 and from the other three estuaries in August 2015 representing monsoon period. The collected samples were immediately transferred to the laboratory for downstream molecular analyses.

### 2.3 Measurement of in situ environmental parameters from Sundarbans estuaries

In situ measurement of environmental parameters such as surface water temperature (digital thermometer, Eurolab ST9269B, Belgium), salinity (Conductivity meter, Erma, Japan), pH (Eco testr, USA), and dissolved oxygen (Eco testr) was conducted in four estuaries of Sundarbans at the time of sampling.

### 2.4 Dissolved nutrient analyses

During sampling, one liter of surface water was also collected from each of the four estuaries of Sundarbans for analyses of dissolved nitrate (Finch et al., 1998), dissolved ortho‐phosphate (Strickland & Parsons, 1972), and dissolved ammonium (Liddicoat, Tibbitts, & Butler, 1975) using a UV‐Vis Spectrophotometer (U2900; Hitachi Corporations, Japan).

### 2.5 DNA extraction from Sundarbans samples

Biomass was concentrated by filtering through 0.22‐µm nitrocellulose filter paper (Pall, USA) following a published protocol (Samanta & Bhadury, 2014). DNA was subsequently extracted using standard published protocol (Bostrӧm, Simu, Hagstrӧm, & Riemann, 2004). Briefly, lysis buffer (50 mM Tris‐HCl, 400 mM NaCl, 20 mM EDTA, 10% SDS, and 750 mM sucrose) (Merck, India) was added to the filter paper and incubated at 50°C for 1 hr. Additional 3‐hr incubation was done after addition of 5 µl of 10 mg/ml Proteinase K (Amresco, USA). The filter paper was further incubated at 37°C for 1 hr after addition of 10 µl of 10 mg/ml Lysozyme (ThermoScientific, USA). DNA was recovered from the aqueous phase following a phenol:chloroform step and precipitated overnight using 3 M sodium acetate (Merck, India) and molecular grade absolute ethanol. DNA was pelleted by centrifugation at 16,000 rcf for 12 min and washed using 70% molecular grade ethanol. The pellet was dissolved in 10 mM Tris‐HCl and run on a 1% agarose gel.

### 2.6 Amplification of bacterial 16S rRNA and Illumina sequencing from Sundarbans samples

Amplification of bacterial 16S rRNA was conducted using barcoded primers Pro340F (5ʹ‐CCTACGGGNBGCASCAG‐3ʹ) and Pro805R (5ʹ‐GACTACNVGGGTATCTAATCC‐3ʹ) (Takahashi, Tomita, Nishioka, Hisada, & Nishijima, 2014). The amplicons with the barcode were subjected to library preparation using NEBNext Ultra DNA Library Preparation kit (NEB, USA). The amplicon library was then purified by 1× AmpureXP beads and checked on Agilent High Sensitivity (HS) chip on Bioanalyzer 2100, quantified in a fluorometer by Qubit dsDNA HS Array Kit (Life Technologies), and loaded onto Illumina MiSeq platform at concentrations of 10–20 pM. The generated sequences have been deposited at the National Centre for Biotechnology Information (NCBI) Short Read Archive data under accession number SRP092508.

### 2.7 Sequence quality control and operational taxonomic unit (OTU) generation

The reads were quality filtered and trimmed by removing adaptor, barcode, and primer sequences. The pair‐end reads were merged together by using Fast Length Adjustment of SHort reads (FLASH) (Magoč & Salzberg, 2011). Chimera sequences were identified by the default method of UCHIME in QIIME (Caporaso et al., 2010; Edgar, Haas, Clemente, Quince, & Knight, 2011). For each dataset, sequences were clustered into operational taxonomic units (OTUs) at 97% sequence identity using UCLUST (Edgar, 2010). The OTUs were classified using RDP Classifier 2.2 at a confidence level of 80% (Wang, Garrity, Tiedje, & Cole, 2007).

### 2.8 Data retrieval from 16S rRNA databases

To compare biogeographic patterns of bacterioplankton communities in Sundarbans estuaries with other global estuaries, 16S rRNA sequences were retrieved from INSDC Sequence Read Archives from the National Centre for Biotechnology Information website (Leinonen, Akhtar, et al., 2011; Leinonen, Sugawara, et al., 2011). Our target was to find bacterioplankton datasets generated from only estuarine surface water. To retrieve bacterioplankton sequences from the Short Sequence Archive (SRA), Boolean search strings were designed and these are summarized in Supporting Information Table S1. Selected datasets were then downloaded using the SRA Toolkit version 2.5.7. Details of sequence data generation are included in Supporting Information Table S2. Bacterioplankton datasets generated over different stations within an estuary were specifically targeted to minimize the effect of local environmental parameters on the overall bacterioplankton community structure representing a particular estuary. The locations of the selected estuaries have been shown in Supporting Information Figure S1.

### 2.9 Comparison of bacterioplankton community structure of estuaries from Sundarbans with other estuaries

Downloaded datasets representing other estuaries were processed similarly to Sundarbans data. Details of data generated from other sites including primers used for amplification, region of the 16S rRNA amplified, and accession number details are summarized in Supporting Information Table S2. The downloaded datasets were generated across multiple studied stations representing the study site. The database contained sequence information from each study station. Such individual sequence files were processed as before. The different data files from each station per estuary were then merged into a single file before further analyses were undertaken. It was assumed that the final merged datasets would contain sequences from all study stations, thereby representing the estuary as a whole. This could essentially eliminated differences in bacterioplankton community composition arising due to local environmental factors. The datasets were normalized to account for the difference in sequence depth from different estuaries. This was performed by rarefying available sequence data to 33,760 sequences, which were the least count among samples based on random subsampling. The SILVAngs analysis pipeline (Quast et al., 2013) was used for taxonomic classification based on the SILVA Reference alignment. The query sequences were first aligned against SILVA SSU rRNA SEED to ensure that all sequences were that of 16S rRNA using SILVA Incremental Aligner v1.2.10 (SINA; Pruesse, Peplies, & Glöckner, 2012). Unclassified sequences, Archaea and chloroplast sequences were removed from downstream analyses. This step also allowed for the removal of low‐quality reads (reads shorted than 50 aligned nucleotides) based on the presence of ambiguous bases (2% of the total length) or homopolymers (2% of the total length). This was followed by a dereplication step where 100% identical reads are marked a replicate. Dereplication was performed using CD‐HIT (v 3.1.2; https://www.bioinformatics.org/cd-hit) applying identity criteria of 1.00 in accurate mode (Li & Godzik, 2006). Pairwise distance estimation and single linkage clustering were used to create clusters of sequences with 97% sequence identity to each other using CD‐HIT. From among each cluster, the longest reads served as a reference sequence which was then taxonomically affiliated to known bacteria using nucleotide BLAST search version 2.2.30+ (Shiryev, Papadopoulos, Schäffer, & Agarwala, 2007) against nonredundant version of SILVA SSU Ref datasets (release 128; Quast et al., 2013). The resulting classification of the reference sequence was mapped to all sequences of the respective cluster and its replicates which provided quantitative information on the number of individual reads per taxonomic assignment. Dominant bacterial phyla were defined as those with abundance of >2% in all samples. The rare bacterial phyla were defined as those that accounted for <0.1% of the total reads (Fuhrman, 2009; Pedrόs‐Aliό, 2006; Sjöstedt et al., 2012). The abundance of bacterial families across different estuaries was plotted on a heat map in excel in order to observe variation in abundance and pattern across estuaries.

### 2.10 Validation of comparison between different hypervariable regions of 16S rRNA

The datasets included in this study used different regions of the 16S rRNA to elucidate bacterioplankton community structure. To validate that different variable regions of the 16S rRNA provide similar taxonomic affiliation, the comparison was done across them. Full‐length 16S rRNA sequences from a previous study conducted in Mooriganga estuary were used for validation (Ghosh & Bhadury, 2018). The full‐length sequences were trimmed to the V2, V3, V4, V1–V2, and V3–V4 variable region combinations corresponding to the variable regions of the metabarcoding datasets. Taxonomic affiliation of the 541 sequences of the individual variable regions and the full length of the 16S rRNA was performed in RDP classifier (Wang et al., 2007). The classification was considered up to the family level at 80% confidence as shown in Supporting Information Table S5. Sequences used for this validation are deposited at GenBank under the accession numbers KX014028–KX014568.

### 2.11 Statistical analyses

The Shannon–Weaver index, Simpson's diversity index, and rarefaction curves were generated at a bacterial family level in Visualization and Analysis of Microbial Population Structures (VAMPS) (Huse et al., 2014). The data were normalized using “Normalized to Percent” option, and values were generated using “Alpha diversity” option under Choose Visualization Method option. The taxonomic depth was set at “family level” for calculation of values. The abundance of bacterioplankton families across the estuaries was normalized and square root transformed. A nonmetric multidimensional scaling (nMDS) ordination was undertaking using Bray–Curtis dissimilarity in PRIMER v6.02 (Clarke & Gorley, 2006). Plots were generated separately for abundant and rare taxa to understand their biogeographic distribution patterns across the estuaries. One‐way ANOVA was performed to compare the abundance of bacterioplankton groups between studied stations (Ståhle & Wold, 1989). Similarity percentage (SIMPER) was performed to identify phyla that contributed most to the dissimilarity between the bacterioplankton community compositions in studied estuaries of Sundarbans (Clarke, 1993). BIO‐ENV was used to explore the effect of measured environmental parameters on changes in the bacterioplankton community structure (Clarke & Ainsworth, 1993). Five environmental parameters, namely surface water temperature, salinity, dissolved oxygen, dissolved nitrate, and dissolved *ortho*‐phosphate available for studied estuaries, were included in the analysis. Environmental variables were normalized, and Euclidean distance between the samples was determined. The OTU abundance data were log (*N* + 1) transformed, and Bray–Curtis similarity coefficient was determined to quantity similarity between bacterioplankton community structures of studied estuaries. Pearson's correlation was undertaken to analyze the influence of environmental parameters on each bacterioplankton group.

## 3 RESULTS

### 3.1 Overview of bacterioplankton community patterns in Sundarbans

**Figure 2:**
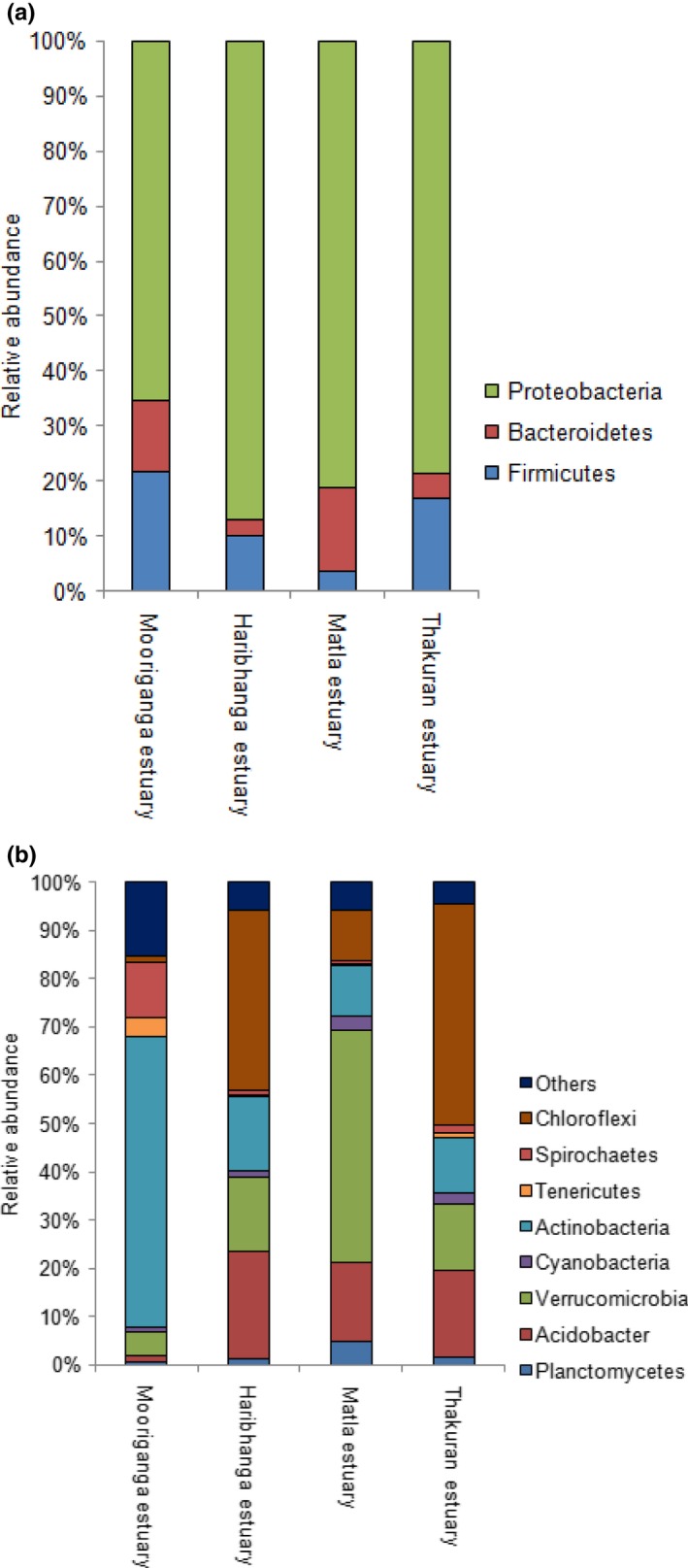
(a) Variation in the distribution of abundant bacterioplankton phyla found in the four estuaries of Sundarbans mangrove ecoregion; (b) variation in the distribution of rare bacterioplankton phyla found in the four estuaries of Sundarbans mangrove ecoregion. Phyla marked as “others” in the graph include WS3, Deferribacteres, Deinococcus‐Thermus, SR1, Lentisphaerae, Chlamydia, Fusobacteria, Nitrospirae, Gemmatimonadetes, OD1, and TM7

Four estuaries located within the Sundarbans were selected, and bacterioplankton community patterns were assessed. The distribution patterns of dominant bacterioplankton phyla and Proteobacterial classes are summarized in Table 1 and shown in Figure 2a. Clustering of OTUs was performed at 97% nucleotide identity. A total of 2,499,148 pair‐end reads were generated from Stn3 representing the Mooriganga estuary which clustered into 55,823 unique OTUs. A total of 215,103 pair‐end reads were generated from StnS1 of Thakuran estuary which clustered into 11,560 unique OTUs. For StnS2 representing the Matla estuary, a total of 320,997 pair‐end reads were generated which clustered into 20,546 OTUs. A total of 242,281 pair‐end reads were generated from StnS3 of Harinbhanga estuary which clustered into 13,887 OTUs.

**Table 1:** Abundance (number of OTUs) of the major bacterioplankton phyla and Protoebacterial classes from the studied estuaries

Bacterioplankton phylum	Delaware Bay	Columbia estuary	Mooriganga estuary	Haribhanga estuary	Matla estuary	Thakuran estuary	Pearl estuary	Jiulong estuary	Hangzhou estuary
Acidobacter	11	380	13	188	296	154	9	168	21
Actinobacteria	1,935	849	543	131	191	97	470	5,103	302
Bacteroidetes	2,919	11,652	7,000	324	2,390	403	888	9,790	867
Cyanobacteria	414	1,169	9	13	54	19	660	3,157	1,656
Firmicutes	43	165	11,931	1,140	550	1,553	58	1,688	163
Planctomycetes	65	223	6	10	88	14	69	650	61
Proteobacteria	8,005	17,692	35,941	9,880	12,639	7,262	2,957	30,057	9,305
Verrucomicrobia	487	887	43	129	863	116	40	496	148

Proteobacterial classes	Columbia estuary	Delaware estuary	Mooriganga estuary	Thakuran estuary	Matla estuary	Harinbhanga estuary	Jiulong estuary	Pearl estuary	Hangzhou estuary
Alphaproteobacteria	3,357	2,096	5	541	1,646	1,942	7,838	1,137	6,136
Betaproteobacteria	2,840	1,219	134	292	569	354	2,236	986	277
Deltaproteobacteria	1,153	91	4	1,550	660	2,868	551	44	16
Epsilonproteobacteria	101	7	5	81	22	31	4,020	13	3
Gammaproteobacteria	4,586	1,370	549	4,398	9,011	3,860	12,668	237	1,438

Across the four estuaries of Sundarbans, an abundance of Proteobacteria was significantly lower in Stn3 (Mooriganga) (one‐way ANOVA, *p* < 0.01), whereas Firmicutes were significantly higher (one‐way ANOVA, *p* < 0.05). The abundance of Cyanobacteria sequences was significantly lower in Stn3 (Mooriganga) (one‐way ANOVA, *p* < 0.1), whereas phyla such as OD1 (9 OTUs) and SR1 (8 OTUs) were higher compared to other stations (one‐way ANOVA, *p* < 0.001). The community level variation was largely contributed by the rare taxa identified from these estuaries. Differences in the distribution pattern of the less abundant or “rare” taxa in these four estuaries are shown in Figure 2b. No significant difference in abundance was found for rare taxa such as OD11, Spirochaetes, and Deferribacteres among the four estuaries of Sundarbans. Interestingly, other rare bacterial phyla such as WS3 (1 OTU) and TM7 (98 OTUs) were identified only from Stn3 (Mooriganga). Abundance of potentially pathogenic bacterial phylum, namely Spirochaetes, was significantly higher in Stn3 (Mooriganga) (101 OTUs) which progressively decreased across stations of the other three estuaries which are further away from human settlement or interference (StnS1 [Thakuran]—15 OTUs, StnS2 [Matla]—10 OTUs, and StnS3 [Harinbhanga]—8 OTUs) (one‐way ANOVA, *p* < 0.05). Interestingly, the total number of unclassified OTUs remained low in studied estuaries of Sundarbans. Only 85 bacterial OTUs (Stn3 [Mooriganga]—20 OTUs, StnS1 [Thakuran]—11 OTUs, StnS2 [Matla]—41 OTUs, and StnS3 [Harinbhanga]—13 OTUs) could not be classified to any taxonomic level beyond kingdom.

### 3.2 Comparison between different variable regions of 16S rRNA

A total of 541 clones were analyzed to check for congruency in taxonomic affiliation based on the selection of different variable regions as indicated in Supporting Information Table S5. Full‐length 16S rRNA sequences (~1,466 bp) were affiliated to known bacterial families using the RDP Classifier (Wang et al., 2007). Only up to family‐level affiliation was considered to maintain parity with the metabarcoding datasets. Individual variable regions namely V2, V3, and V4 and combination of variable regions namely V1–V2, V1–V3 and V3–V4 were trimmed out from the full‐length sequences. Comparison of each variable region revealed that 86% of the sequences (464/541) showed identical taxonomic affiliation to the family level. Sequences belonging to families such as Prolixibacteraceae, Campylobacteraceae, Marinilabiliaceae, and Cryomorphaceae showed different taxonomic affiliation when assigned using different variable regions of 16S rRNA. The obtained taxonomic affiliation of each variable region and the full‐length 16S rRNA is shown in Supporting Information Table S5.

### 3.3 Biogeographic patterns of bacterioplankton taxa across studied global estuaries

**Figure 3:**
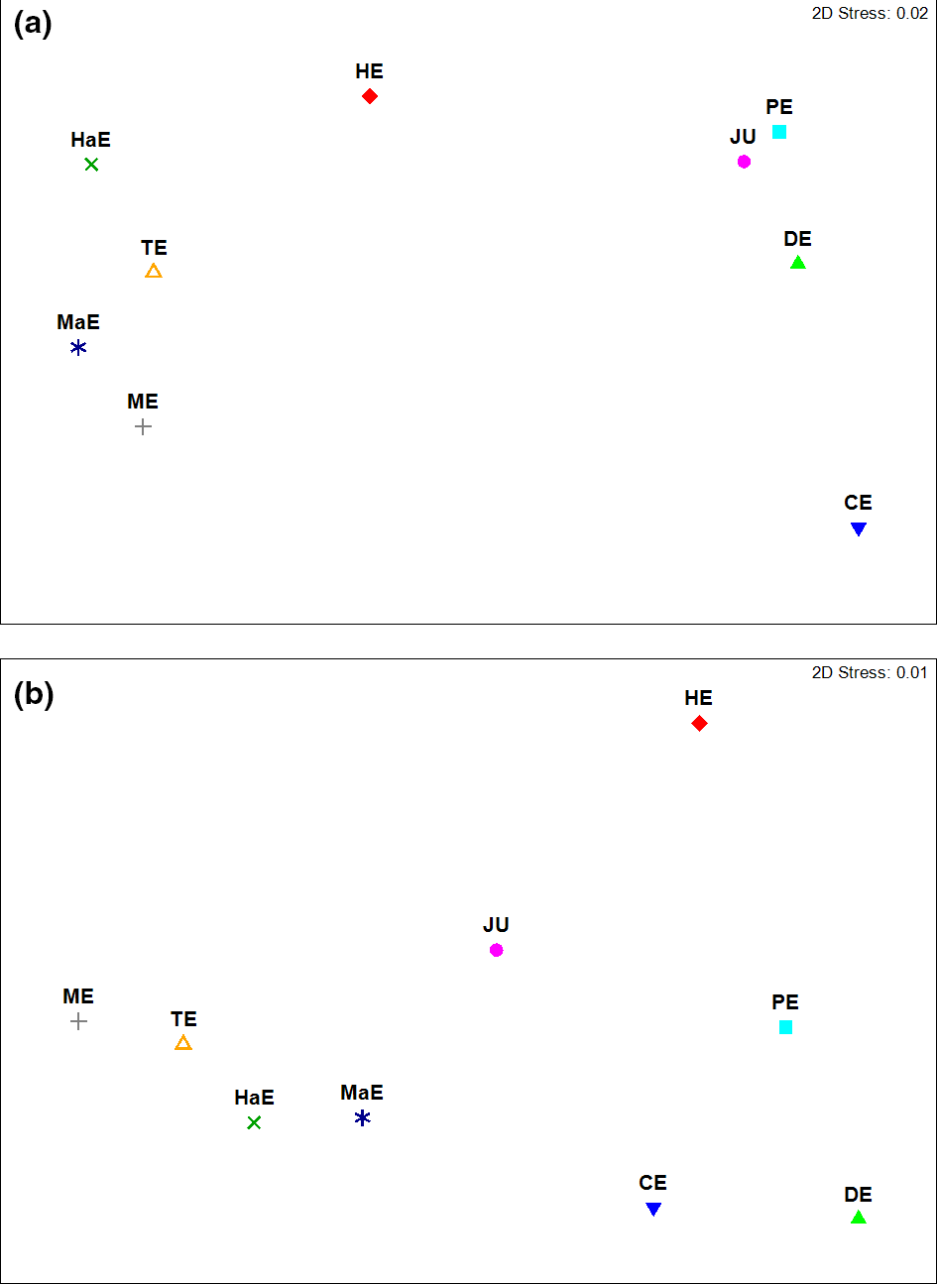
(a) Nonmetric multidimensional scaling (nMDS) ordination plot showing clustering of abundant bacterioplankton across the studied estuaries. (b) Nonmetric multidimensional scaling (nMDS) ordination plot showing clustering of rare bacterioplankton across the studied estuaries. The abbreviations represent the names of the estuaries (CE: Columbia estuary; DE: Delaware estuary; HaE: Harinbhanga estuary; HE: Hangzhou estuary; JE: Jiulong estuary; MaE: Matla estuary; ME: Mooriganga estuary; PE: Pearl estuary; TE: Thakuran estuary)

In order to identify bacterioplankton biogeographic patterns, we compared bacterioplankton communities of studied estuaries in Sundarbans to that of other global estuaries. The nMDS ordination plot of the abundant taxa indicated two distinct clusters. The estuaries of Sundarbans namely Mooriganga, Thakuran, Matla, and Harinbhanga formed one cluster. The second cluster was formed by Delaware, Pearl, and Jiulong estuaries (Figure 3a). Unlike the abundant taxa, the nMDS ordination plot of the rare taxa did not show distinct clusters (Figure 3b). The estuaries located within the Sundarbans mangrove ecoregion showed average dissimilarity between 9.42% and 18.60% as seen by SIMPER analysis (Supporting Information Table S3). The observed average dissimilarity between studied estuaries of Sundarbans is accounted by the abundant bacterioplankton phyla including Acidobacteria, Actinobacteria, Bacteroidetes, Cyanobacteria, Proteobacteria, and Verrucomicrobia.

Proteobacteria was the dominant and most widespread phylum identified and comprised >50% of the total abundance of bacterioplankton sequences across all estuaries. Proteobacteria sequences were recorded in highest abundance in Harinbhanga estuary of Sundarbans (relative abundance—81%) and abundance was lowest in Columbia estuary (relative abundance—52%). Undoubtedly, Proteobacteria was the most abundant phylum across all the estuaries, but there was significant variation in the abundance of Proteobacterial classes in these estuaries. Gammaproteobacteria and Alphaproteobacteria were found to be the dominant members of bacterioplankton communities which accounted for ~44% of total bacterioplankton abundance in all studied estuaries. Gammaproteobacteria was the most abundant class in estuaries of Sundarbans, Jiulong, and Columbia. The abundance varied from 49% in Matla estuary to only 5% in Pearl estuary. A similar variation was observed in terms of the abundance of Alphaproteobacteria. The bacterioplankton community structure of Delaware, Hangzhou, and Pearl estuaries was dominated by Alphaproteobacteria. Among the estuaries of Sundarbans, Mooriganga estuary recorded highest abundance of Alphaproteobacteria (relative abundance—42%), whereas lowest was recorded in Thakuran estuary (relative abundance—5%). Interestingly, Firmicutes sequences constituted a significantly high proportion of the bacterioplankton community in terms of abundance in estuaries of Sundarbans (one‐way ANOVA, *p* < 0.05). Apart from the estuaries in Sundarbans, Firmicutes did not constitute an abundant member of bacterioplankton community in any of the other studied estuaries. Of all the known classes of Firmicutes, only Bacilli, Clostridia, and Erysipelotrichia were encountered in estuaries of Sundarbans. Clostridia were the major class of Firmicutes encountered in Sundarbans estuaries, whereas only 1% of the bacterioplankton community comprised of Bacilli. The estuaries of Sundarbans harbored a high abundance of Clostridia, which was otherwise found in low abundance in the other estuaries. Yet another phylum such as Cyanobacteria which comprised nearly 13% of the bacterioplankton community of Pearl and Hangzhou estuaries appeared in low abundance in the other estuaries.

At the family level, the overwhelming abundance of Hydrogenophilaceae was encountered in the estuaries of Sundarbans, as well as in Jiulong and Columbia estuaries. Coriobacteriaceae (phylum—Actinobacteria), known to normally dwell on a mammalian body, and Erysipelotrichaceae (phylum—Firmicutes) were found in high abundance only in Mooriganga, Pearl, and Jiulong estuaries.

**Figure 4:**
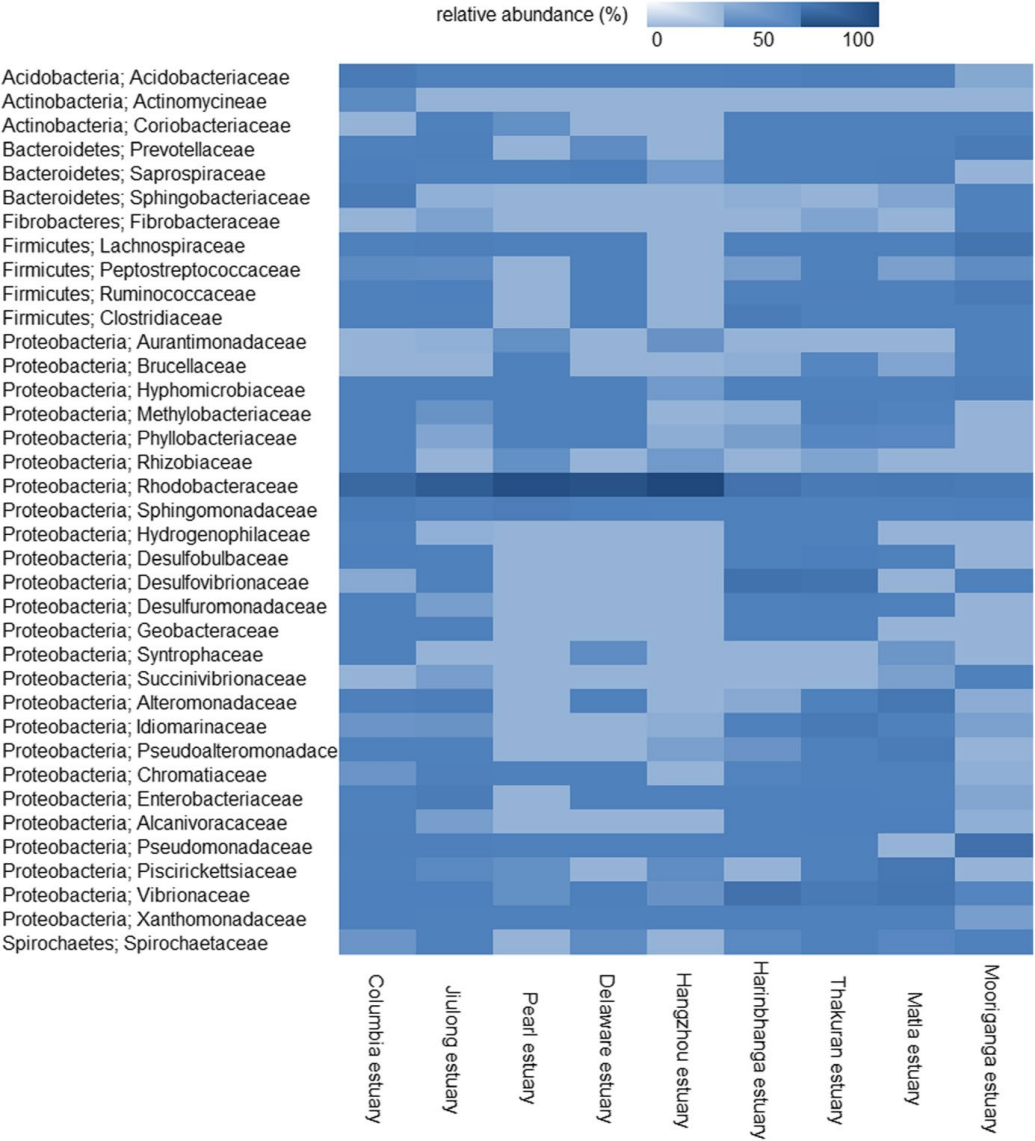
Heat map shows the distribution of bacterioplankton communities in the studied estuaries. The colors represent the abundance of the particular bacterial family at the study site. It represents the distribution pattern of each bacterial family across the studied estuaries. The phylum and class of each bacterioplankton family are shown on the right‐hand side of the heat map

The most abundant bacterioplankton families were found to be ubiquitous as evident from Figure 4. In comparison with the dominant phyla which showed a more widespread distribution across all studied estuaries, rare phyla were found to be unique to specific estuaries. The abundance of Chloroflexi in Harinbhanga and Thakuran estuaries constituted 3% and 4% of the total abundance of bacterioplankton, whereas this phylum was nearly undetected in the other estuaries. Rare phyla including Lentisphaerae, Nitrospira, and TM7 showed presence in all the studied estuaries with insignificant variation in abundance. Interestingly, Planctomycetes and Spirochaetes constituted 1% of the total bacterioplankton population of Pearl, Columbia, Jiulong, and Matla. The estuaries appeared to share the similar abundance of bacterial phyla including Chlamydia, Fusobacteria, OD1, SR1, and Thermotogae. Each of this phylum was further resolved to class and family levels. Though the trend remained similar between phylum and class levels, dominant families showed interesting trends. Only three bacterial families belonging to the rare phyla namely Caldilineales, Anaerolineae (phylum—Chloroflexi), and Nitrospiraceae (phylum—Nitrospirae) were found in all the estuaries. Bacterial families such as Anaeroplasmataceae (phylum—Tenericutes), Fibrobacteraceae (phylum—Fibrobacteres), and Deinococcus (phylum—Deinococcus‐Thermus) were found to be exclusive to estuaries of Sundarbans. Yet, other families such as Spiroplasmataceae (phylum—Tenericutes) and Veillonellaceae (phylum—Firmicutes) were exclusive to Delaware estuary and Parachlamydiaceae (phylum—Chlamydiae) were encountered only in Jiulong estuary. Moreover, as indicated by the heat map in Figure 4, a clear segregation was observed for bacterial families including Rhodobacteriaceae, Sphingomonadaceae, Acidobacteriaceae, Vibrionaceae, and Xanthomonadaceae which showed widespread presence in comparable abundance across studied estuaries. At the same time, bacterial families such as Desulfomuromonadaceae, Idiomarinaceae, and Hyphomicrobiaceae indicated dispersed distribution across studied estuaries.

Rarefaction analysis was undertaken to compare relative OTUs richness among targeted estuaries as shown in Supporting Information Figure S2. The rarefaction curves showed saturation for all the estuaries indicating optimum NGS sequencing effort (Supporting Information Figure S2). The Shannon–Weaver values were found to be highest in Matla estuary (3.68) and lowest in Pearl estuary (2.57) (Supporting Information Table S4). Simpson's index value of studied estuaries also showed similar trends (Supporting Information Table S4).

### 3.4 Influence of environmental parameters on bacterioplankton biogeography

The available data representing environmental parameters and dissolved nutrients present in estuaries have been summarized in Table 2. No significant difference in the environmental parameters was found among studied estuaries using one‐way ANOVA (*p* = 0.79). The observed trends in bacterioplankton community patterns were found to correlate with existing environmental parameters. All the abundant bacterial phyla identified in this study including Acidobacteria, Actinobacteria, Bacteroidetes, Planctomycetes, Proteobacteria, and Verrucomicrobia were strongly influenced by surface water temperature (*p* < 0.001). Planctomycetes and Firmicutes were positively correlated with salinity, whereas Verrucomicrobia and Acidobacteria showed a negative correlation with salinity (*p* < 0.05). Interestingly, several of the abundant phyla including Bacteroidetes, Proteobacteria, Firmicutes, and Verrucomicrobia showed a strong correlation with dissolved ortho‐phosphate concentration (*p* < 0.001). This trend was also clearly reflected at the bacterial class levels. The observed patterns of Proteobacterial classes including Gammaproteobacteria, Betaproteobacteria, Deltaproteobacteria, and Epsilonproteobacteria appeared to be influenced by surface water temperature, whereas Alphaproteobacterial abundance did not show any correlation with surface water temperature. Alphaproteobacteria, Betaproteobacteria, and Deltaproteobacteria showed a strong correlation with dissolved ortho‐phosphate concentration (*p* < 0.001). Only, Epsilonproteobacteria showed correlation with salinity (*p* < 0.001). Other abundant bacterial classes such as Clostridia, Bacteroidia, Sphingobacteria, and Actinobacteria were strongly influenced by surface water temperature and dissolved ortho‐phosphate concentration (*p* < 0.001). The rare taxa identified in this study showed a strong correlation with surface water temperature and salinity. Three bacterial phyla namely OD10, OD11, and WS3 showed a strong correlation with dissolved nitrate concentration (*p* < 0.05). The observed trends were further reconfirmed by BIO‐ENV analysis which highlighted that surface water temperature and dissolved nutrients such as dissolved nitrate and ortho‐phosphate to be the primary determinant in controlling observed biogeographic patterns of bacterioplankton communities across studied estuaries (BIOENV *ρ_w_* = 0.907).

**Table 2:** Details of environmental parameters recorded in the different estuaries considered in this study

Site	Filtration technique	Salinity	Surface water temperature (°C)	Dissolved oxygen (mg/L)	Total dissolved nitrate (µM)	Total dissolved ortho‐phosphate (µM)
Columbia estuary	0.22 µm	3.9	19.4	4.95	12.7	0.5
Delaware Bay	0.8 µm followed by 0.22	15.07	23.95	NA	41.95	0.81
Jiulong estuary	0.22 µm	26	28.69	NA	20.27	1.39
Pearl estuary	3 µm followed by 0.22 µm	17.92	28.26	6.68	56.1	0.92
Zhejiang Bay	0.22 µm	29.2	NA	6.88	10.499	0.379
Sundarbans mangrove ecoregion	0.22 µm	10	30	3.4	29	3.4

## 4 DISCUSSION

Transitional environments such as estuaries witness a gradient in salinity, temperature, and dissolved nutrients owing to continuous mixing of freshwater and saline water (Crump, Adams, Hobbie, & Kling, 2007; Fortunato, Herfort, Zuber, Baptista, & Crump, 2012). Fast‐flowing rivers bring down a huge amount of sediments and dissolved nutrients which are washed down its entire course ultimately ending up in the coast. Moreover, estuaries present in highly human populated areas tend to see a large input of nitrogen and phosphorus including from agriculture‐ and aquaculture‐related activities (Lacerda, 2006). These changing physico‐chemical conditions can act as stressors for resident microbial communities (Paerl, Valdes, Peierls, Adolf, & Harding, 2006; Sun et al., 2012). In coastal ecosystems such as mangroves, plant matter serves as an additional source of organic matter in nearby aquatic systems (Dittmar et al., 2006). Plant matter such as mangrove litterfall contributes to 30%–60% of total primary production (Bunt, Boto, & Boto, 1979). Litterfall into the water results in rapid release of dissolved organic matter (DOM) and tannins (Fell, Cefalu, Masters, & Statzell‐Tallman, 1975; Newell, Fell, Statzell‐Tallman, Miller, & Cefalu, 1984; Robertson, 1988; Steinke, Holland, & Singh, 1993). Decomposition of remaining particulate organic matter is a slow process that is largely driven by microbial communities (Robertson, Alongi, & Boto, 1992). Previous studies have shown that greater structural and functional versatility in diverse communities may facilitate exploitation of environmental resources to a further extent (Jolliffe, 1997). It has been suggested that under varying environmental conditions, greater species diversity could help to maintain the productivity of ecosystems by stabilization of environmental conditions (Yachi and Loreau, 1999).

Numerous studies have been undertaken to elucidate bacterial community structures from various estuaries globally. These extensive datasets of millions of reads using next‐generation sequencing technologies are available in public databases such as GenBank/DDBJ/ENA (Benson, Karsch‐Mizrachi, Lipman, Ostell, & Wheeler, 2005; Leinonen, Akhtar, et al., 2011; Leinonen, Sugawara, et al., 2011; Mashima et al., 2017). The 16S rRNA is commonly used as a chronometer for this purpose (Brown & Fuhrman, 2005; Clarridge, 2004; Martínez‐Murcia, Antόn, & Rodríguez‐Valera, 1999). Owing to short read lengths generated in NGS such as those generated by Illumina platforms, studies focus on sequencing only one or two variable regions of the 16S rRNA. The accuracy of different hypervariable regions of the 16S rRNA in taxonomic assignment has been long debated as there is no clear consensus on the region of 16S rRNA to be used for sequencing. Huse et al. (2008) investigated the accuracy of V3 and V6 regions in comparison the with full‐length 16S rRNA in determining taxonomy of generated sequences. They found the V3 region to be 99% accurate for assignment to genus level, whereas the V6 is 97% accurate. These regions have been found to be consistent for the elucidation of rare taxa (Huse et al., 2008). Bacterioplankton communities considered in this study were elucidated using a combination of V1, V2, V3, and V4 hypervariable regions of the 16S rRNA (Bartram, Lynch, Stearns, Moreno‐Hagelsieb, & Neufeld, 2011; Kircher & Kelso, 2010; Vilo & Dong, 2012). Numerous studies have discussed the possible effect of use of different variable regions in addition to primer selection to be a critical factor in elucidating bacterioplankton community structure (Hamp, Jones, & Fodor, 2009; Liu, Lee, Vanlare, Kasper, & Mazmanian, 2008; Mizrahi‐Man, Davenport, & Gilad, 2013; Soergel, Neelendu, Knight, & Brenner, 2012; Sundquist et al., 2007; Wu, Hartman, Ward, & Eisen, 2008). However, the efficiency of different primer sets or use of different variable regions to accurately resolve the bacterioplankton communities of estuaries was beyond the scope of this study. The efficiency of different variable regions of the 16S rRNA in providing similar taxonomic resolution at least to the family level could be validated using a simple step. This has also been observed in a detailed study by Wear, Wilbanks, Nelson, and Carlson (2018) where the authors clearly show that the choice of primers does not greatly affect bacterioplankton community composition. Additionally, the authors also validated using large datasets that qualitative information of bacterioplankton communities generated by use of different primers is highly comparable.

Due to almost negligible anthropogenic interference, estuaries of Sundarbans represent near pristine conditions (Dhame, Kumar, Ramanathan, & Chaudhuri, 2016; Mandal, Ray, & Ghosh, 2012; Samanta & Bhadury, 2016). Clustering pattern showed the bacterioplankton communities of Sundarbans formed a distinct cluster for both abundant and rare phyla. Our results clearly show for the first time that estuaries of Sundarbans are overwhelmingly dominated by Proteobacteria and thus are important players in mediating coastal ecosystem processes. The high abundance of Firmicutes in Mooriganga estuary could be attributed to the shallow depth and high load of suspended particulate matter (SPM) (Choudhury et al., 2015). On the other hand, other three estuaries in Sundarbans are exclusively fed by saline water, and therefore, mixing of the sediment in the water column is much less compared to Mooriganga which have resulted in a low abundance of sediment‐associated bacterioplankton such as Firmicutes. Moreover, members of this phylum can break down DOM (Taketani, Dias, & Andreote, 2010), which could indicate a possible role of Firmicutes in breaking down DOM from mangrove litterfall. A high abundance of Firmicutes in the Columbia estuary was expected due to the presence of estuarine turbidity maximum (ETM); however, the abundance of Firmicutes was as low as that observed for the other estuaries. Interestingly, in all the other estuaries targeted as part of this study, Firmicutes sequences were found to very low (0%–1%) indicating that mixing and resulting suspension of sediment may not be the only major contributing factor. The abundance of Actinobacteria sequences was found to be significantly higher in Mooriganga estuary. This could be due to the availability of complex organic substances in estuarine water surrounding Mooriganga some of which could be anthropogenic in origin and thus promoting their cell abundance (Choudhury et al., 2015; Vilo & Dong, 2012). It is already well known that this group can degrade a range of polymeric substances such as cellulose, lignin, chitin, starch, and laminarin (Linos et al., 2002; Pranamuda, Chollakup, & Tokiwa, 1999) which could also originate from mangrove plants. Interestingly, high abundance of Actinobacteria has been reported from other mangrove environments such as those located in Brazil (Silveira et al., 2011). We also encountered higher abundance of Verrucomicrobia sequences in Matla estuary. However, this group was not encountered in Mooriganga estuary which highlights the spatial differences in bacterioplankton biogeography across Sundarbans within a localized geographic scale. Verrucomicrobia is known for dependence on organic matter and can be important in carbon cycling (Canfora et al., 2014). Incidentally, organic matter load in Matla estuary could be high due to litterfall as this part of the Sundarbans has relatively higher density and diversity of mangrove plants (Mukherjee & Ray, 2012). More recently, it has been reported that Verrucomicrobia play important role in the assimilation of dissolved inorganic carbon in coastal water (DeLorenzo et al., 2012). Although Verrucomicrobia sequences did not constitute more than 3% in other global estuaries, nevertheless their presence in almost all the estuaries indicates functional significance. For the first time, we show that Verrucomicrobia sequences are ubiquitous in global estuarine water given that they are known to be widely present in soil environment and also have reported from other coastal ecosystems but not from estuaries (Freitas et al., 2012).

Other bacterial phyla, which can be considered “rare” in terms of the abundance of sequence reads in the studied estuaries, include Chloroflexi, Spirochaetes, Nitrospirae, Tenericutes, and Deinococcus‐Thermus. Out of these rare phyla, Deinococcus‐Thermus sequences were not encountered in Delaware and Hangzhou estuaries. These minor components of bacterioplankton communities could be functionally more relevant or may harbor an important repertoire of stress‐related genes (Freitas et al., 2012). It is well established that some members of Nitrospirae, Chloroflexi, and Proteobacteria are nitrite‐oxidizing bacteria (NOB) which catalyzes nitrification and thus play important roles in nitrogen cycling (Sorokin et al., 2012; Wang et al., 2015). Interestingly, all the four estuaries of Sundarbans and Columbia estuary showed a high abundance of Nitrospirae and Chloroflexi sequences compared to other studied estuaries although dissolved nitrate concentration was not higher in the former. However, the possibility of a “lag phase” between the increase in abundance of NOB and high dissolved nitrate concentrations could be a possibility. The concentration of dissolved nitrate in Delaware (41.95 µM) and Pearl estuaries (56.1 µM) was found to be higher than the other studied estuaries, but interestingly NOB abundance was low in both of these estuaries. Tenericutes sequences were found in all the estuaries, except Hangzhou estuary, with the highest abundance in Mooriganga estuary. Studies have shown a correlation of Tenericutes with Spirochaetes and hypothesized a possible symbiotic or parasite role between these two groups (Canfora et al., 2014) although such correlation was not encountered. From the viewpoint of nitrogen cycling, Deferribacteres sequences were encountered in all the four estuaries of Sundarbans, in addition to Delaware estuary and Jiulong estuary. Chloroflexi were encountered in almost all the studied estuaries except Hangzhou estuary. The phylum Chloroflexi which has a phototrophic lifestyle is frequently reported from the hypersaline environment (Ma & Gong, 2013), but we did not find any correlation with available salinity datasets as part of our study. In fact, the highest abundance of Chloroflexi was recorded from Columbia estuary which also had the lowest salinity among all estuaries targeted as part of this study. Such site specificity is further reinstated by the nMDS ordination plot of rare taxa that does not indicate distinct clusters.

Low abundance of Cyanobacteria was encountered in studied estuaries, except for Columbia estuary where it was relatively higher (relative abundance—3%). The observed low abundance of Cyanobacteria in studied estuaries of Sundarbans may be due to high suspended particulate matter in the water column which can affect light penetration (Choudhury et al., 2015; Litchman, 2003). Therefore, limiting light could be an important factor that controls the abundance of Cyanobacteria in studied estuaries. It has been also reported in the literature that *Synechococcus* population have the ability to tolerate low oxygen in water (Nogales, Lanfranconi, Piña‐Villalonga, & Bosch, 2011). In the Mooriganga estuary of Sundarbans, *Synechococcous* populations are present throughout the year (Singh & Bhadury, 2018) even when dissolved oxygen concentration has been found to be low during summer (Bhattacharjee et al., 2013).

Broad biogeographic patterns of bacterioplankton communities observed at the phylum level were also reflected at the class level in studied estuaries. Bacterial classes such as Gammaproteobacteria, Alphaproteobacteria, and Clostridia were widely encountered in all the studied estuaries. As evident from this study, all the four estuaries of Sundarbans and Columbia estuary were dominated by Gammaproteobacteria. The observed pattern is in line with a study conducted earlier in the coastal water of Hong Kong (Zhang, Liu, Lau, Ki, & Qian, 2007). The authors showed the higher dominance of Gammaproteobacteria (with a high proportion of Oceanospirillales, Alteromondales, Enterobacteriales, and Vibrionales) over those of Alphaproteobacteria based on 16S rRNA clone library and sequencing approaches. Alphaproteobacteria usually constitute the dominant bacterial class in coastal water (Kirchman et al., 2005; Pommier et al., 2007; Zhang et al., 2007; Zhang, Lau, Ki, Thiyagarajan, & Qian, 2009). Additionally, it is known that the marine end of estuaries (higher salinity zones) are dominated by Alphaproteobacteria that otherwise normally exist in low abundance in low salinity water (Bouvier & del Giorgio, 2002; Cottrell & Kirchman, 2003). The high abundance of Gammaproteobacteria in the estuaries of Sundarbans and Columbia estuary is intriguing given that salinity was relatively higher than other estuaries. Williams et al. (2013) demonstrated the growth of marine Gammaproteobacteria (mainly Oceanospirillales and Alteromondales) in the presence of algal organic matter degrading Flavobacteria in the surface water of coastal East Antarctica. But incidentally, Flavobacteria was not found in high abundance in the estuaries of Sundarbans (<1%) but represented 25% of the total abundance in Columbia estuary. Hence, overwhelming dominance of Gammaproteobacteria from Sundarbans cannot be explained by one single factor but could be due to multiple factors such as the role of other bacterial groups and affinity for substrates. Sequences representing Pseudomonadaceae and Enterobacteriaceae were encountered from studied estuaries, and their number was found to be much higher in estuaries of Sundarbans. Incidentally, the presence of polyaromatic hydrocarbon in sediment and water of Sundarbans can form an excellent substrate for members of Pseudomonadaceae and Enterobacteriaceae (Hesham, Mawad, Mostafa, & Shoreit, 2014; Sarma, Bhattacharya, Krishnan, & Lal, 2004). Moreover, sequences representing Succinivibrionaceae were also encountered in low number from Jiulong, Mooriganga, and Matla estuaries. Succinivibrionaceae, a group of strict anaerobes and reported mainly from gastro‐intestinal tract of animals (Tamames, Abellán, Pignatelli, Camacho, & Moya, 2010), are capable of fermenting glucose and other carbohydrates to succinate and acetate can also utilize DOMpool (Kersters et al., 2006). Such groups indicate the presence of DOM and also hint toward the pristine nature of the Sundarbans mangrove ecosystem. Similarity in abundance of such bacterial families could be resulting in the distinct clustering of the Sundarbans estuaries as observed in the nMDS ordination plot.

A common understanding of the “distance‐decay” relationship is that bacterial community similarity decreases with increasing geographic distance. The distance–decay relationship directly challenges the more popular concept of “everything is everywhere” for bacteria. Our study shows that geographically distant estuaries share a high degree of similarity which seems to override temporal variations commonly observed in bacterioplankton communities. We observed clear biogeographic patterns of bacterioplankton communities which can be explained by prevailing environmental conditions in studied estuaries. The influence of environmental parameters on the bacterioplankton communities of the studied sites was in line with previously published literature (Fortunato et al., 2012; Lozupone & Knight, 2007). We found surface water temperature and dissolved nutrients such as dissolved nitrate and ortho‐phosphate to strongly influence observed patterns of bacterioplankton communities (BIOENV *ρ_w_* = 0.907). Consistent distribution patterns of dominant members of the bacterioplankton communities could be also due to the similarity in surface water temperature prevailing across studied estuaries. At the same time, no single environmental parameter could account for the observed biogeographic pattern of rare bacterioplankton taxa across studied estuaries. At the bacterioplankton biogeography level, we find many bacterial classes showing ubiquitous distribution across estuaries globally. It is important to mention that although ubiquitous trends were observed, the studied estuaries were not sampled across same time point. While the estuaries of Sundarbans were sampled during the Indian monsoon, the NGS data for Pearl, Jiulong, and Hangzhou estuaries were also generated previously during the monsoon season of Southern China. However, NGS datasets of Columbia and Delaware estuaries were obtained from samples collected during late winter and early summer of Northern Hemisphere. Therefore, it may be possible that some of the seasonal trends of bacterioplankton communities may not be reflected in the studied datasets. However, despite this seasonal variability, our study shows remarkable similarity in terms of structure and function of bacterioplankton communities.

Biogeography studies allow the retrieval of bacterioplankton groups widespread in all study sites along with site‐specific groups. It may be difficult to determine whether or not this estuary‐specific bacterioplankton are related to particular stress prevalent in each estuary. The observed biogeographic patterns of bacterioplankton across global scale can help understand their role in ecosystem‐level processes such as utilization of DOM pool, autotrophic, and heterotrophic production as well as carbon and nitrogen cycling (Cavigelli & Robertson, 2000; Horz, Barbrook, Field, & Bohannan, 2004; Naeem & Li, 1997; van der Heijden et al., 1998). Furthermore, such biogeographic patterns yield information on bacterioplankton co‐occurrence which could ultimately help toward developing culture‐based approaches. Information about substrate requirement and essential growth conditions can be understood as found in this study and applied for the establishment of bacterioplankton cultures, especially those within mangrove ecosystem. Given there is increasing anthropogenic pressure, nevertheless the ecosystem level health of studied estuaries was good as bacterioplankton indicative of eutrophication or of urban origin was found to be infrequent in terms of abundance based on the analyzed dataset. To conclude, this study which incorporated robust bioinformatics analysis of generated as well as available NGS datasets provided an interesting glimpse of bacterioplankton communities across local, regional, and global scales.

## CONFLICT OF INTEREST

The authors declare no conflict of interest.

## AUTHORS CONTRIBUTION

PB and AG conceived the idea; AG undertook experiments and data analysis; AG and PB wrote the manuscript. All authors read and approved the final manuscript.

## ETHICS STATEMENT

No human and/or experimental animal subjects were involved in this study.

## DATA ACCESSIBILITY

The datasets supporting the conclusions of this article are available in the National Centre for Biotechnology Information (NCBI) Short Read Archive data under accession number SRP092508, https://www.ncbi.nlm.nih.gov/sra. The datasets of the other estuaries used in this study are publicly available for download, and the corresponding accession numbers of the datasets are provided in the manuscript. All environmental data generated and analyzed in this table are provided in the manuscript.
