# Supplementary figures and images for: Exploring biogeographic patterns of bacterioplankton communities across global estuaries
Source: Microbiologyopen. 2018 Oct 10;8(5):e00741. doi: 10.1002/mbo3.741 (PMC6528645; doi:10.1002/mbo3.741)

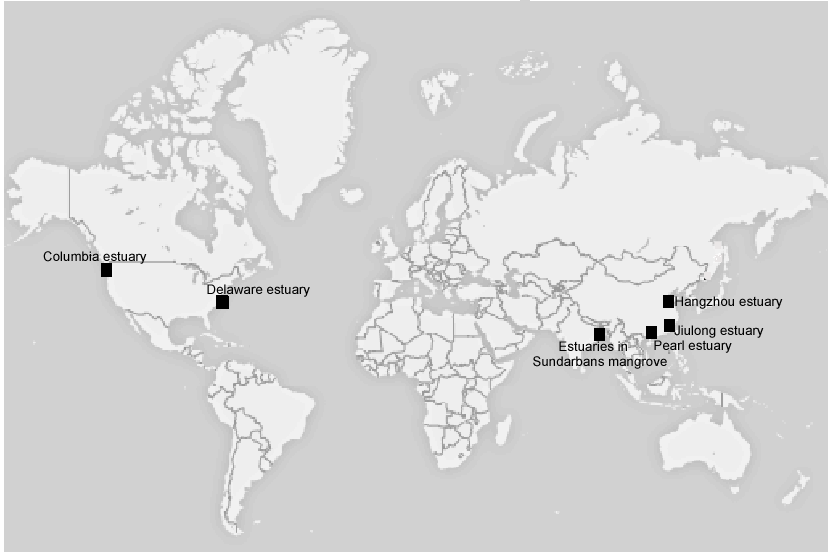

Supplement: Supplementary file 1 [file MBO3-8-e00741-s001.tif]

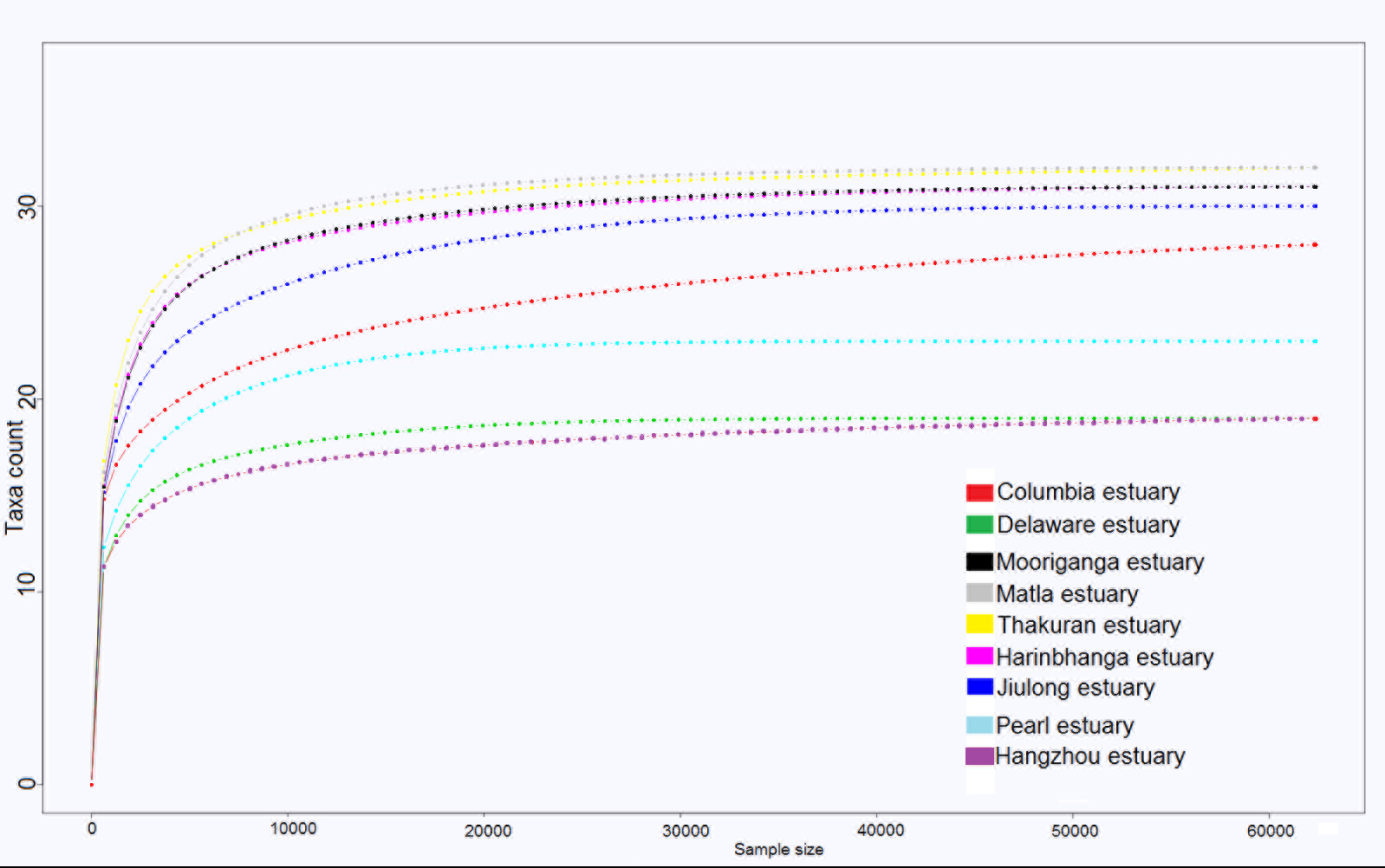

Supplement: Supplementary file 2 [file MBO3-8-e00741-s002.tif]
