# Supplementary material for: Exploring biogeographic patterns of bacterioplankton communities across global estuaries
Source: Microbiologyopen. 2018 Oct 10;8(5):e00741. doi: 10.1002/mbo3.741 (PMC6528645; doi:10.1002/mbo3.741)
Supplement: Supplementary file 3 [file MBO3-8-e00741-s003.docx]

| **Table S1:** Boolean search strings used to retrieve bacterioplankton datasets from SRA as of August, 2016 | |  |
| --- | --- | --- |
| **Target group** | **Search string^1^** | **No. of datasets available in SRA** |
| Search for bacteria | ((bacteria[organism]) OR prokaryote [organism]) NOT archaea | 319219 |
| Search for 16S rRNA sequences | 16S rDNA OR 16S rRNA OR 16S ribosomal RNA gene OR 16S small subunit ribosomal RNA OR small subunit ribosomal RNA gene OR 16S rRNA gene | 142257 |
|  |  | (3000 are marine metagenome) |
|  |  | (2208 are aquatic metagenome) |
|  |  | (1936 are freshwater metagenome) |
| Bacteria from mangrove ecosystems | (mangrove bacteria[text word] OR mangrove bacteria [title]) | No search result |
| Bacterioplankton from estuary | (((estuary[text word]) OR estuary[title]) AND bacteria [text word] AND bacteria [title]) | 20 |
| Search for specific mangrove ecosystems | e.g. (Brazil mangrove bacteria [text word]) OR Brazil mangrove bacteria [title] | No search result |
|  |  |  |
|  | (florida mangrove) AND bacteria |  |
|  |  |  |
|  | ((florida mangrove [text word] OR florida mangrove [title]) AND bacteria |  |
| ^1^OR, NOT, AND are Boolean operators in uppercase format | |  |
