# Supplementary material for: Exploring biogeographic patterns of bacterioplankton communities across global estuaries
Source: Microbiologyopen. 2018 Oct 10;8(5):e00741. doi: 10.1002/mbo3.741 (PMC6528645; doi:10.1002/mbo3.741)
Supplement: Supplementary file 4 [file MBO3-8-e00741-s004.docx]

**Table S2:** Details of data on bacterioplankton communities generated in other estuaries

| Study site and data accession number in database | Region of 16S rRNA gene amplified | 16S rRNA primer used | Study stations | Accession number | Reference |
| --- | --- | --- | --- | --- | --- |
| Columbia estuary  SRP006412  ENA | V2 | 27F and 338R | New_plume  lat: 46.239, long: -124.161 | ERS709857 | Fortunato et al. 2013 |
|  |  |  | Old_plume  lat: 46.233, long: -124.16 | ERS709859 |  |
| Delaware estuary  SRA052537  SRA | V1-V3 | 28F and 519R | 20  lat: 39.2201, long: -75.2705 | SRR495182 | Campbell and Kirchman, 2013 |
|  |  |  | 22  lat: 36.152, long: -75.1657 | SRR495186 in |  |
|  |  |  | 24  lat: 39.0507, long: -75.1095 | SRR495193 |  |
|  |  |  | 26  lat: 39.0507, long: -75.1095 | SRR495198 |  |
| Jiulong estuary  SRP044039  SRA | V3-V4 | Bakt_341F and Bakt_805R | S8 | SRR1508521 | Shan et al. 2015 |
| Pearl estuary  SRP019932  SRA | V1-V3 | 28F and 519R | A08 | SRR1014068 | Liu et al. 2015 |
|  |  |  | C2 | SRR1014070 |  |
|  |  |  | C3 | SRR1014072 |  |
|  |  |  | F412 | SRR1014074 |  |
|  |  |  | F414 | SRR1014076 |  |
| Hangzhou estuary  DRA002865  In DDBJ | V4 | F515 and R806 | 10  lat: 30.69, long: 121.74 | DRR028674 | Wang et al. 2015 |
|  |  |  | 11  lat: 30.68, long: 121.89 | DRR028684 |  |
|  |  |  | 12  lat: 30.68, long: 121.56 | DRR028694 |  |
|  |  |  | 20  lat: 30.62, long: 121.9 | DRR028720 |  |
|  |  |  | 21  lat: 30.6, long: 121.6 | DRR028721 |  |
|  |  |  | 24  lat: 30.57, long: 121.25 | DRR028724 |  |
|  |  |  | 25  lat: 30.55, long: 121.28 | DRR028725 |  |
|  |  |  | 27  lat: 30.5, long: 121.78 | DRR028726 |  |
|  |  |  | 28  lat: 30.5, long: 121.78 | DRR028727 |  |
|  |  |  | 33  lat: 30.42, long: 121.28 | DRR028729 |  |
|  |  |  | 36  lat: 30.4, long: 121.35 | DRR02873 |  |
|  |  |  | 42  lat: 30.37, long: 121.63 | DRR028734 |  |
