# Supplementary material for: Exploring biogeographic patterns of bacterioplankton communities across global estuaries
Source: Microbiologyopen. 2018 Oct 10;8(5):e00741. doi: 10.1002/mbo3.741 (PMC6528645; doi:10.1002/mbo3.741)
Supplement: Supplementary file 5 [file MBO3-8-e00741-s005.docx]

**Table S3:** Average dissimilarity between the bacterioplankton communities representing each of the studied estuaries as obtained from SIMPER analysis

| Average dissimilarity | Columbia  estuary | Mooriganga  estuary | Matla  estuary | Thakuran estuary | Harinbhanga  estuary | Pearl  estuary | Jiulong  estuary | Hangzhou estuary |
| --- | --- | --- | --- | --- | --- | --- | --- | --- |
| Delaware estuary | 17.3 | 41.15 | 37.03 | 39.64 | 36.69 | 11.86 | 9.24 | 28.34 |
| Columbia  estuary |  | 45.67 | 41.17 | 43.34 | 43.56 | 21.79 | 20.01 | 32.42 |
| Mooriganga  estuary |  |  | 18.6 | 14.92 | 22.33 | 40.53 | 36.36 | 32.9 |
| Matla  estuary |  |  |  | 15.44 | 16.01 | 39.05 | 34.7 | 23.19 |
| Thakuran estuary |  |  |  |  | 9.46 | 39.35 | 35.09 | 24.43 |
| Harinbhanga  estuary |  |  |  |  |  | 39.37 | 35.75 | 21.99 |
| Pearl  estuary |  |  |  |  |  |  | 7.07 | 18.29 |
| Jiulong  estuary |  |  |  |  |  |  |  | 23.99 |
