# Supplementary material for: Exploring biogeographic patterns of bacterioplankton communities across global estuaries
Source: Microbiologyopen. 2018 Oct 10;8(5):e00741. doi: 10.1002/mbo3.741 (PMC6528645; doi:10.1002/mbo3.741)
Supplement: Supplementary file 6 [file MBO3-8-e00741-s006.docx]

| **Table S4:** Details of number of OTUs and alpha-diversity values (bacterial family level)  found in all estuaries considered in this study | | | | |
| --- | --- | --- | --- | --- |
| Study site | Number of OTUs  (pre-normalized) | Shannon-Weaver index | Simpson index |  |
|  |  |  |  |  |
| Columbia estuary | 62400 | 2.94 | 0.87 |  |
| Delaware Bay | 5462 | 2.77 | 0.89 |  |
| Mooriganga estuary | 30474 | 3.2 | 0.92 |  |
| Thakuran estuary | 11560 | 3.51 | 0.9 |  |
| Matla estuary | 20546 | 3.68 | 0.95 |  |
| Harinbhanga estuary | 13887 | 3.2 | 0.92 |  |
| Jiulong estuary | 48366 | 3.43 | 0.94 |  |
| Pearl estuary | 1701 | 2.57 | 0.8 |  |
| Hangzhou Bay | 27348 | 2.66 | 0.88 |  |
